# Comparison of biomechanical analysis results using different musculoskeletal models for children with cerebral palsy

**DOI:** 10.3389/fbioe.2023.1217918

**Published:** 2023-09-26

**Authors:** Zhibo Jing, Jianda Han, Juanjuan Zhang

**Affiliations:** ^1^ Tianjin Key Laboratory of Intelligent Robotics, Nankai University, Tianjin, China; ^2^ Institute of Robotics and Automatic Information System, Nankai University, Tianjin, China; ^3^ College of Artificial Intelligence, Nankai University, Tianjin, China; ^4^ Smart Sensing Interdisciplinary Science Center, Nankai University, Tianjin, China

**Keywords:** locomotion biomechanics, musculoskeletal models choice, consistency, opensim, cerebral palsy

## Abstract

**Introduction:** Musculoskeletal model-based simulations have gained popularity as a tool for analyzing human movement biomechanics. However, when examining the same gait, different models with varying anatomical data and assumptions may produce inconsistent biomechanical results. This inconsistency is particularly relevant for children with cerebral palsy, who often exhibit multiple pathological gait patterns that can impact model outputs.

**Methods:** The aim of this study was to investigate the effect of selecting musculoskeletal models on the biomechanical analysis results in children with cerebral palsy. Gait data were collected from multiple participants at slow, medium, and fast velocities. Joint kinematics, joint dynamics, and muscle activation were calculated using six popular musculoskeletal models within a biomechanical simulation environment.

**Results:** The degree of inconsistency, measured as the root-mean-square deviation, in kinematic and kinetic results produced by the different models ranged from 4% to 40% joint motion range and 0%–28% joint moment range, respectively. The correlation between the results of the different models (both kinematic and kinetic) was good (R > 0.85, *P* < 0.01), with a stronger correlation observed in the kinetic results. Four of the six models showed a positive correlation between the simulated muscle activation of rectus femoris and the surface EMG, while all models exhibited a positive correlation between the activation of medial gastrocnemius and the surface EMG (*P* < 0.01).

**Discussion:** These results provide insights into the consistency of model results, factors influencing consistency, characteristics of each model’s outputs, mechanisms underlying these characteristics, and feasible applications for each model. By elucidating the impact of model selection on biomechanical analysis outcomes, this study advances the field’s understanding of musculoskeletal modeling and its implications for clinical gait analysis model decision-making in children with cerebral palsy.

## 1 Introduction

Biomechanical analysis plays an important role in forecasting the impact of musculoskeletal injury on gait, developing rehabilitation devices for patients with pathological gaits, and understanding the biomechanics of human movement. Musculoskeletal models are widely used in motion biomechanical analysis (Shippen and May 2010; Wagner et al., 2010; Steele et al., 2012; Amiri and Bull, 2022), particularly in estimating quantities that are challenging to be measured noninvasively (e.g., muscle force (Li et al., 2022; Luis et al., 2022), joint contact force (Hosseini Nasab et al., 2022), joint torque (Heinrich et al., 2022)) and predicting the influence of external forces (e.g., exoskeleton assistance) or gait conditions (e.g., rough terrain) on human motion.

Musculoskeletal models of specific joints, segments, and the whole body have been built by researchers. In general, such models are comprised of bones with three-dimensional (3D) geometries, joints with kinematics definitions, and muscles with force generation characteristics. The parameters of muscle tendon units were usually determined based on autopsy data and nuclear magnetic resonance imaging data. The models of lower limb joints (Wickiewicz et al., 1984; Yamaguchi and Zajac, 1989; Friederich and Brand, 1990) are widely used in musculoskeletal models which were developed and deployed in biomechanical simulation environments such as OpenSim (Delp et al., 2007) and ANYBODY (Damsgaard et al., 2006). Researchers can access source codes of the models and extend the work of other people easily. Users can use the models for biomechanical analysis without the ability to develop them.

Biomechanical analysis of children with cerebral palsy (CP) has been widely conducted with musculoskeletal models. CP patients often suffer from pathological gaits such as equinus, crouch, and excessive hip flexion (Wren et al., 2005). Researchers used models to study the causes and effects of pathological gaits: changes in muscle force and tibiofemoral contact force with increased knee flexion (Steele et al., 2012), effects of crouch gait on hip-knee muscle extension during single-limb stance (Hicks et al., 2008), and contributions of muscles to centroid acceleration and joint angular acceleration in the squat gait (Steele et al., 2010). These studies scaled the generic musculoskeletal models developed from adult cadaver data using the children measure. This procedure ignored the patient-specific geometry and parameters of muscles and bones. In order to generate a more personalized model, researchers tried to generate a musculoskeletal model from medical images (Kainz et al., 2021). Due to the lack of resources (i.e., hardware conditions, maturity of methods, and tolerance of children) in clinic, it is difficult to collect necessary data and generate completely subject-specific models. Therefore, it is a common practice in clinic to use the scaled general musculoskeletal model.

The same analyses performed with different models or software are expected to produce consistent results. However, several studies (Sandholm et al., 2011; Wagner et al., 2013; Trinler et al., 2019; Weinhandl and Bennett, 2019) showed differences in joint angles, joint torques, muscle forces, muscle moment levers, and tibial contact forces produced by different models and primarily discussed causes of the differences. While there were some issues to be considered: comparisons of too few models, no collection of participants’ data, and few studies of patients with abnormal gaits. Some important fundamental issues, such as the effect of walking speed on the consistency of model output, the characteristics and mechanisms of each model output, and which model has the highest correlation between simulated muscle activation and measured surface EMG of children with cerebral palsy, are not yet fully understood.

The aim of this study was to investigate the effects of model choices on the outputs of biomechanical analysis, consistency between the outputs of different models, and factors affecting the consistency in children with cerebral palsy. We collected patient data and used a biomechanical analysis method based on musculoskeletal models to calculate joint kinematics, kinetics, and muscle activation in a simulation environment. We defined and calculated the degree of inconsistency between the model outputs and analysed factors affecting the consistency. We tried to relate the differences in results to the underlying modelling and computational assumptions. We hope to help physicians or researchers understand the characteristics of musculoskeletal model results and select the appropriate model for their own research.

## 2 Methods

### 2.1 Musculoskeletal models

This study included six generic three-dimensional full-body musculoskeletal models (Gait2354 (Delp et al., 1990), Gait2392 (Delp et al., 1990), Lai’s model (Lai et al., 2017), Falisse’s model (Falisse et al., 2019), Rajagopal2015 model (Rajagopal et al., 2016), RUN model (Hamner et al., 2010), see Table 1) in OpenSim. Although Gait2392 and Gait2354 did not have upper limbs, they can still be used as full-body models when focusing on lower limb movements. The two models adopted the lower extremity joint of Delp et al. (1990), the low back joints and anthropometry of Anderson and Pandy (1999), and the planar knee model of Yamaguchi and Zajac (1989). Falisse’s model and RUN Model were modifications and extensions of the Gait 2392 model. Rajagopal2015 model and Lai’s model were derived from the lower body model published by Arnold et al. (2010). Since Arnold’s model only had lower limbs, it was not included in this study.

**TABLE 1:** Numbers of DOFs and muscles of the models.

	M1	M2	M3	M4	M5	M6
Model name	Gait2354	Gait2392	Falisse’s model	Run model	Rajagopal2015 model	Lai’s model
Lower limb DoF	14	14	12	10	14	14
Upper limb DoF	0	0	8	10	14	14
Torso DoF	9	9	9	9	9	9
Total DoF	23	23	29	29	37	37
Muscles number	54	92	92	92	80	80

These models had different numbers of degrees of freedom (DoFs, see Table 2). Both Gait2392 and Gait2354 had 23 DoFs. They had 6 DoFs between the pelvis and the ground. The lumbar, hip, knee, ankle and subtalar joints had 3, 3, 1, 2 and 1 DoFs respectively. Compared to Gait2392 and Gait2354, Falisse’s model added bilateral arms with 4 DoFs per arm and removed the metatarsal joint, so it had 29 degrees of freedom in total. Although the number of RUN model was also 29, each arm had 5 DoFs, and it did not have subtalar and metatarsal joints. Both Rajagopal2015 model and Lai’s model had 37 DoFs, and each had 7 DoFs for each upper arm and lower limb.

**TABLE 2:** Participants’ information.

Participant	Gender	Height	Body Mass	Age	Slow speed	Medium speed	Fast speed
		(m)	(kg)	(yrs)	(m/s)	(m/s)	(m/s)
1	female	1.38	35	10	0.30	0.50	0.75
2	female	1.19	21	5	0.30	0.55	0.80
3	male	1.31	28	6	0.30	0.50	0.70
4	male	1.09	18	5	0.30	0.55	0.75
5	male	1.26	27	8	0.30	0.40	0.55
6	male	1.04	17	5	0.10	0.20	0.35

Gait2354 reduced 92 muscles in Gait2392 to 54 muscles. Gait2354 increased the maximum isometric force of each muscle and modified other muscle and tendon parameters accordingly to compensate for the lack of strength caused by the reduction in the number of muscles. The muscle numbers, muscle-tendon parameters, and muscle geometry of Falisse’s model and RUN model were the same as those in Gait2392. Rajagopal2015 had 80 muscle-tendon units, and the muscle force distribution was updated based on a young, healthy population. Based on Rajagopal 2015, Lai et al. updated the knee joint muscle path and some muscle force parameters.

### 2.2 Experimental setup

Six children with cerebral palsy were recruited (age 6.50 ± 2.07 years; weight 24.33 ± 6.92 kg; height 1.21 ± 0.13 m; and mean ± S.D., see Table 2) for walking experiments in Human Robot Interactive Gait Lab of Nankai University. The study was approved by the Ethics Committee of Nankai University. All participants were provided with written informed consent before completing the protocol.

**FIGURE 1:**
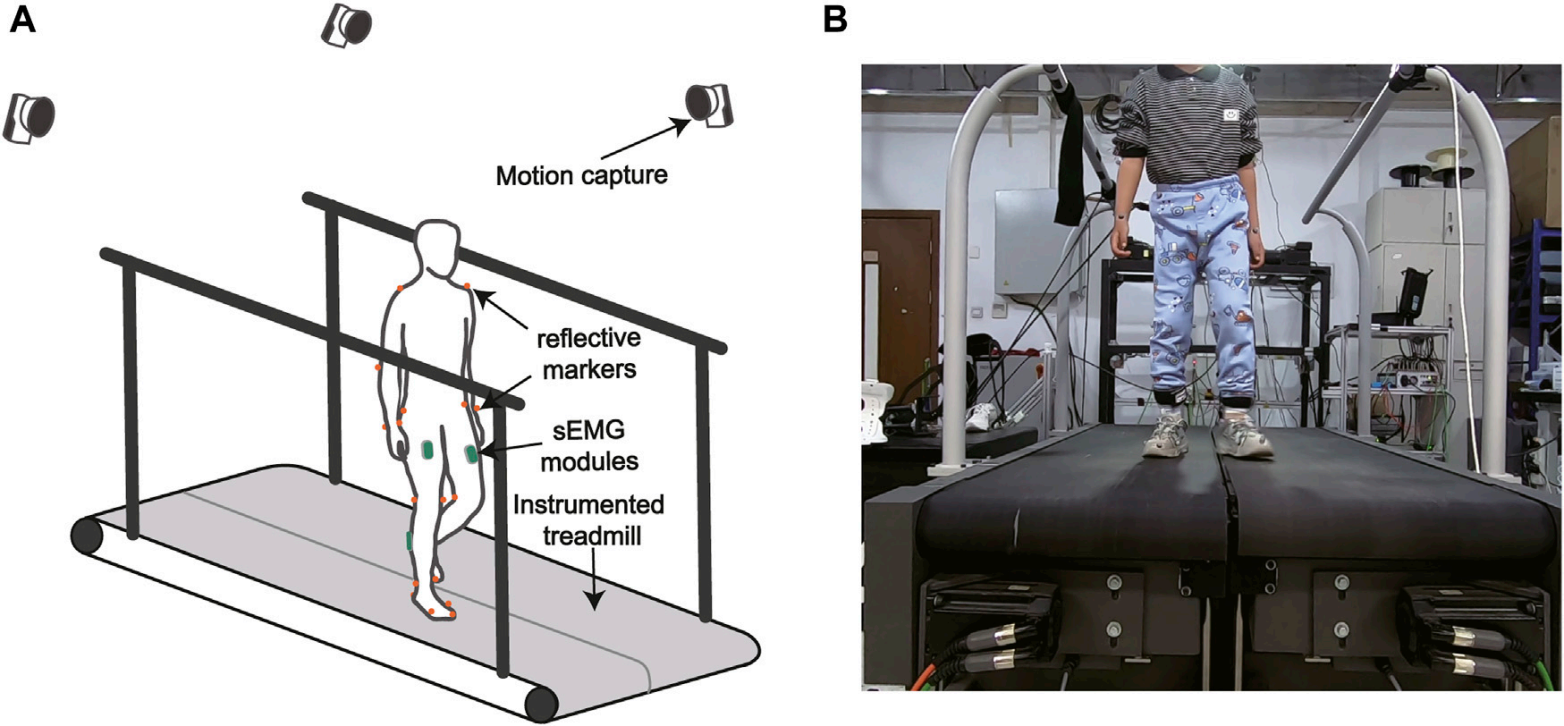
**(A)** The gait analysis hardware system. **(B)** The experimental setup. Muscle surface EMG was recorded by wireless EMG modules. Ground reaction forces were measured by a force-plate instrumented treadmill. A motion capture system recorded reflective marker trajectories.

Four sEMG modules (Bagnoli, Delsys, MA, United States) were placed on the medial gastrocnemius (m.GAS) and the rectus femoris (RF) of participants’ bilateral legs respectively to record the sEMG signals during locomotion at a frequency of 500 Hz (see Figure 1). Raw sEMG signals were high-pass filtered with a second-order Butterworth filter (cutoff frequency 20 Hz), full-wave rectified, and low-pass filtered with a second-order Butterworth filter (cutoff frequency 10 Hz). Twenty nine optical reflective markers were placed on the anatomical landmarks and functional joint centers of participants. A motion capture system (Oqus 700+, Qualisys, Gothenburg, Sweden) with nine camera lenses was used to record trajectories of markers at a sampling rate of 100 Hz. We used Qualisys Track Manager to fill and smooth the marker trajectories. Ground reaction forces (including Fx, Fy, Fz, Mx, My, and Mz) were measured at a sampling rate of 500 Hz by a force-plate instrumented treadmill (FIT, Bertec Corporation, OH, United States). Raw forces and torque were low-pass filtered with a second-order Butterworth filter (cutoff frequency 12 Hz).

In the experiments, the subjects first wore sensors and held the still standing posture which was the default pose of the musculoskeletal models we set for at least 10 s. The motion capture system recorded the positions of markers in the static pose for scaling of the generic musculoskeletal models. The treadmill velocity then increased from 0.3 m/s to the maximum velocity that the patient could bear, in increments of 0.05 m/s. Subjects walked at each walking velocity for at least 30 s. The data of the first stable step in each velocity for each subject was used for subsequent analysis.

### 2.3 Biomechanical analysis

We performed model-based biomechanical analysis in Matlab R2021b by calling the API of Opensim 4.1, including scaling with the scale tool, inverse kinematics with the IK tool, inverse dynamics with the residual reduction algorithm (RRA) tool, and simulated muscle activation with the computed muscle control (CMC) tool (Thelen and Anderson, 2006). Each segment of the models was first scaled to match the subject’s anthropometric measurements. The scale factors were ratios of the distances of marker pairs in the static experiments to the corresponding virtual distances in the model. Body mass and muscle parameters related to length were also scaled according to these ratios. Joint coordinate values corresponding to the static pose were computed. Marker positions were adjusted at the same time to match experimental marker locations. For each scaled model, the IK tool was used to solve for the joint angles to minimize the differences between the experimentally measured marker positions and the virtual positions. The RRA tool computed joint dynamics from kinematics and measured ground reaction forces and reduced the magnitude of the residual force by slightly adjusting joint kinematics and model mass properties. Joint torque was normalized to body weight and was presented in unit of *N* ⋅ *m*/*kg* ^−1^. The CMC tool was used to generate a set of muscle activations, resulting in a coordinated muscle-actuated simulation of the subject’s movements. In order to study the effect of different models on the results, all procedures and parameter settings were the same on all models.

### 2.4 Data analysis

To quantify the consistency between the biomechanical results yielded by different models, we averaged the joint angle waveforms yielded by six models for each participant at each velocity trial, and calculated the root-mean-square deviation (RMSD) between the joint angle waveform yielded by each model and the averaged waveform. Then we normalized the RMSD to the ranges of joint angles. The RMSD and the normalized RMSD of the joint torque were calculated using the same method.

We calculated the average and range of joint angles (represented as $\bar{\theta }$ and ⌊*θ*⌉, respectively), and the average and range of joint moments (represented as $\bar{\tau }$ and ⌊*τ*⌉, respectively) for each participant for the entire gait cycle using each model at each velocity trial. Then we calculated the means of $\bar{\theta }$ at each velocity trial (represented as $\bar{\theta }$ -MAV), the means of $\bar{\tau }$ at each velocity trial (represented as $\bar{\tau }$ -MAV), the means of ⌊*θ*⌉ at each velocity trial (represented as ⌊*θ*⌉-MAV), and the means of ⌊*τ*⌉ at each velocity trial (represented as ⌊*τ*⌉-MAV) for each participant using each model. Finally, we calculated the means of $\bar{\theta }$ -MAV across participants (represented as $\bar{\theta }$ -MAV-MAP), the means of $\bar{\tau }$ -MAV across participants (represented as $\bar{\tau }$ -MAV-MAP), the means of ⌊*θ*⌉-MAV across participants (represented as ⌊*θ*⌉-MAV-MAP), and the means of ⌊*τ*⌉-MAV across participants (represented as ⌊*τ*⌉-MAV-MAP) with each model.

We performed correlation analyses on results of the same biomechanics yield by different models. The correlation coefficients between the ankle angle of each participant yielded by each model at each velocity trial and the ankle angle of that participant yielded by the other models at that velocity trial. We calculated these correlation coefficients in the kinematic and kinetic results of all joints. The correlation coefficients between the muscle activation yielded models and the measured sEMG values in the medial gastrocnemius and the rectus femoris for all participants at each velocity trial. All statistical analyses were conducted with MATLAB R2021b (MathWorks, Natick, MA, United States).

## 3 Results

The RMSDs and the normalized RMSDs for kinematic results ranged from 4% to 40% and from 1.5° to 7.3° respectively (shown in Tables 3, 4), and the RMSDs and the normalized RMSDs for dynamic results ranged from 0% to 28% and from 1.2 × 10^−2^ *N* ⋅ *m*/*kg* to 19.8 × 10^−2^ *N* ⋅ *m*/*kg* respectively (shown in Tables 5, 6). For ankle kinematics, the normalized RMSDs were the biggest during the slow walking trials, and those were the smallest during the fast walking trials. A similar pattern was observed for knee and hip kinematics. However, the RMSDs of kinematics were similar during different velocity walking trials. The normalized RMSDs of dynamics were smaller than those of kinematics as a whole. No obvious change trend was observed for the normalized RMSDs of dynamics during different velocity walking trials.

**TABLE 3:** Normalized RMSD of kinematics of participants on all joints.

Participant	ankle (%)			knee (%)			hip (%)		
	Slow	Normal	Fast	Slow	Normal	Fast	Slow	Normal	Fast
1	17	11	17	6	4	3	13	10	7
2	23	15	11	7	5	5	24	16	16
3	11	10	8	5	4	4	8	9	8
4	20	13	12	12	11	9	6	6	4
5	10	6	4	7	8	7	13	11	9
6	40	21	11	16	15	9	13	13	9
mean	20	13	11	9	8	6	13	11	9

**TABLE 4:** The RMSD of kinematics of participants on all joints.

Participant	ankle (°)			knee (°)			hip (°)		
	Slow	Normal	Fast	Slow	Normal	Fast	Slow	Normal	Fast
1	5.2	5.2	5.4	2.2	2.2	2.1	3.8	3.7	3.7
2	4.5	4.7	4.3	4.0	3.6	3.4	7.3	6.8	6.5
3	2.5	2.6	2.7	2.5	2.4	2.2	2.6	2.7	2.8
4	2.9	3.3	3.2	5.9	5.1	5.4	2.1	2.2	1.9
5	1.9	1.5	1.6	2.7	2.3	3.0	3.5	3.1	3.2
6	3.3	3.2	3.2	4.8	4.7	4.7	2.1	2.2	2.6

**TABLE 5:** The normalized RMSD of dynamics of participants on all joints.

Participant	Ankle (%)			Knee (%)			Hip (%)		
	Slow	Normal	Fast	Slow	Normal	Fast	Slow	Normal	Fast
1	3	2	2	8	7	4	4	3	3
2	3	2	1	11	5	5	5	4	3
3	1	1	1	1	1	1	1	2	1
4	4	2	3	20	14	12	6	4	4
5	20	23	8	26	27	14	11	28	11
6	1	1	1	4	2	2	1	0	1
Mean	5	5	3	12	9	6	5	7	4

**TABLE 6:** The RMSD of dynamics of participants on all joints.

Participant	Ankle × 10^–2^(*N* ⋅ *m*/*kg*)			Knee × 10^–2^(*N* ⋅ *m*/*kg*)			Hip × 10^–2^(*N* ⋅ *m*/*kg*)		
	Slow	Normal	Fast	Slow	Normal	Fast	Slow	Normal	Fast
1	1.9	1.9	2.1	3.4	3.4	3.2	3.3	2.6	3.0
2	2.3	1.9	2.1	4.5	3.4	3.6	3.9	3.5	3.4
3	1.8	3.5	1.8	4.2	6.0	3.8	4.1	6.5	4.4
4	3.2	1.8	3.7	6.9	3.7	6.1	3.7	2.5	3.7
5	9.3	18.2	10.6	5.7	5.9	8.6	7.2	19.8	9.0
6	4.9	3.1	4.3	13.2	7.7	7.8	2.4	1.2	2.5

**FIGURE 2:**
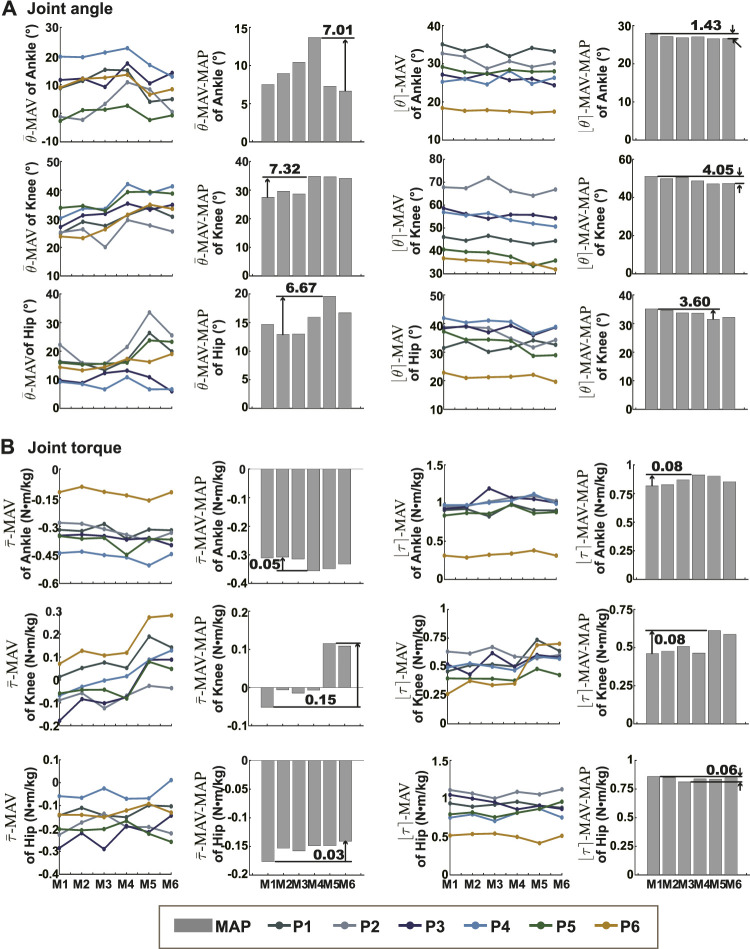
The means and ranges of joint kinematics and dynamics. **(A)** Showed the $\bar{\theta }-\text{MAV}$ , $\bar{\theta }-\text{MAV}-\text{MAP}$ , ⌊*θ*⌉-MAV, and ⌊*θ*⌉-MAV-MAP of all joints in the sagittal plane. The six colored lines represented $\bar{\theta }-\text{MAV}$ and ⌊*θ*⌉-MAV of six participants with the six models. Grey bar charts showed $\bar{\theta }-\text{MAV}-\text{MAP}$ and ⌊*θ*⌉-MAV-MAP with the six models. **(B)** Showed the same features value of joint torque.

The means and the ranges ( $\bar{\theta }$ -MAV, $\bar{\theta }$ -MAV-MAP, ⌊*θ*⌉-MAV, and ⌊*θ*⌉-MAV-MAP) of different joints were shown in Figure 2. The maximal differences of the means (51.3%, 21.0%, 34.1%, 14.0%, 130.3%, and 17.0%) were bigger than those of the ranges (5.1%, 8.0%, 10.3%, 8.8%, 13.1%, and 7.0%). The means of ankle angle, knee angle, and knee torque appeared in a certain order. The means of ankle angle yielded by model M4 were the biggest in five participant results. The means of knee angle yielded by model M4-M6 were bigger than those yielded by model M1-M3 in five participant results. The means of knee torque with model M1 were the lowest in four participants, and those with model M5 were the largest in four participants. The means of knee torque with model M2, model M3, and model M4 were similar.

**FIGURE 3:**
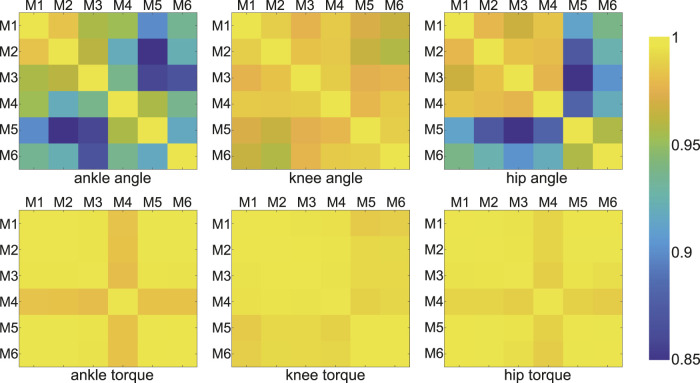
Correlation coefficients of joint angles and moments between any two models (among all models). Each color block showed the coefficient corresponding to the two model results. The coefficients were all greater than 0.85 (P < 0.01), and the color of them from 0.85 to 1 was shown in the color bar on the right.

Correlation coefficients between outputs (dynamics and kinematics) yielded by different models on all joints were all greater than 0.85 (P < 0.01, Figure 3). In general, the correlation between dynamics was stronger than that between kinematics. The coefficients of knee angle between models were greater than 0.95 (p < 0.01), but a few coefficients of ankle and hip were smaller than 0.9 (p < 0.01). The correlations between the hip joint angles obtained using the M1-M4 models were better than the correlations between those and the results from other models.

**FIGURE 4:**
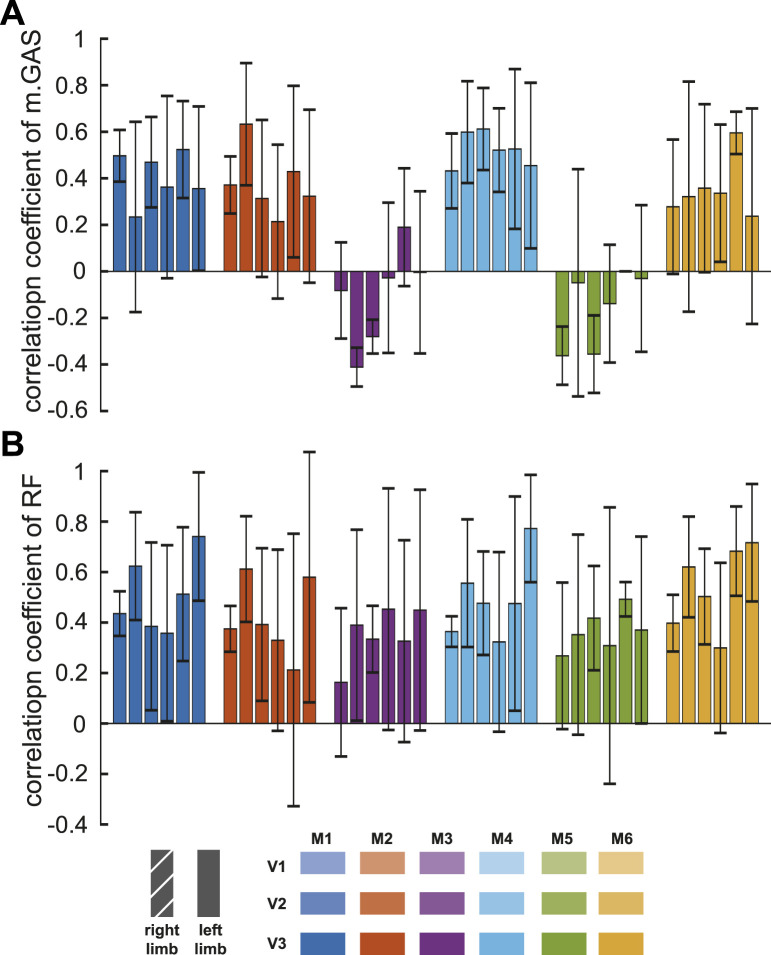
Correlation coefficients between simulated muscle activation yielded by the models and measured sEMG of muscles [medial gastrocnemius, **(A)**, rectus femoris, **(B)**]. The six colors represented outputs of different models at three velocities. Each bar showed the mean and one standard deviation of the six participants.

Except for model M3 and model M5, measured RF sEMG and muscle activity computed by the models showed positive correlations, and the correlation coefficients of them were less than 0.6 (P < 0.05, Figure 4). Measured m. GAS sEMG and the muscle activity computed by all models were positively correlated, and the correlation coefficients of them were less than 0.8 (P < 0.05).

## 4 Discussion

The purpose of this study was to investigate differences between the biomechanical results yielded by six popular models during different velocity walking trials. To achieve it, various kinds of data were collected from six children with cerebral palsy while walking on a treadmill, and biomechanical analyses were performed on each participant using all six models. Differences in joint kinematics, dynamics, and muscle activations were observed in the results with different models.

Consistency and accuracy in results across models are the ultimate goals in biomechanical analysis. They are generally explored in comparative studies of musculoskeletal models. Although consistent outputs are unnecessarily accurate, inconsistent outputs of at least some, maybe not all, models are inaccurate. Due to the unavailability of the actual value, we discussed the difference between models (consistency) but not the difference between outputs and actual values (accuracy). In this paper, the RMSD and the normalized RMSD were calculated to represent the consistency in results across models.

Our reported results of the RMSD (kinematics: 1.5°–7.3° and kinetics: 1.2 × 10^−2^ *N* ⋅ *m*/*kg*-19.8 × 10^−2^ *N* ⋅ *m*/*kg*) were similar to the results of Kainz et al. (kinematics: below 5° and kinetics: 0.15 *N* ⋅ *m*/*kg*) (Kainz et al., 2021), which were between generic-scaled and MRI-based models, and the results of Flux et al. (kinematics: 3°–8°) (Flux et al., 2020), which were between the Human Body Model and conventional gait models. For joint kinematics, the normalized RMSD decreased with increasing velocity, indicating that the kinematic results of the different models were relatively more consistent at high walking speeds. This was because the RMSDs were almost constant with speed, while joint range of motion increased significantly with speed.

The possible reason for the wide variation in the RMSDs across patients was the variation in gait between patients. There was a wide distribution of age, weight, height, cerebral palsy subtype, and degree of pathological gait among the patients participating in this study, so there were large differences in gait between patients. Gait characteristics have an impact on the consistency of the results, for example, Wagner et al. (2013) found better agreement of moment arms and tibiofemoral joint contact forces occurring at low knee flexion angles. We also observed a similar effect of gait on consistency: participant P5 had the smallest ankle angle of anyone during the walking trials, and his consistency of ankle moment results was the worst of anyone.

The magnitude of the differences between the model results varied across joints. One study (Falisse et al., 2018) compared the outputs of the Gait2392 and Human Body Models and showed that the largest differences in kinematics and kinetics occurred at the hip joint. While, our results showed that the largest normalized RMSD between model outputs for kinematics was in the ankle joint and the largest normalized RMSD between kinetics was in the knee joint. The largest differences in the mean and range values of the kinematic and kinetic results output by the different models were in the knee joint. The reason for the different conclusions may be that the two studies calculated the differences differently and the models compared were different.

The output values produced by the different models (e.g., ankle joint angle) showed the same order in multiple subjects. Although it was not known which was closer to the true value, it helped us understand whether each model’s output was skewed larger or smaller across the six models. The differences in definitions and parameters of the models were partly responsible for difference of the outputs. The planar knee model specified the transformation between the femur, tibia, and patella reference frames as a function of knee angle. The functions were based on Yamaguchi et al. (Yamaguchi and Zajac, 1989) (Gait2354, Gait2392, and Falisse’s model), Seth et al. (2010) (Run model), and Walker et al. (1988) (Rajagopal2015 model and Lai’s model). The larger $\bar{\theta }-\text{MAV}$ of knee outputted by the latter three models indicated that the functions they used resulted in greater joint flexion.

The difference of the model outputs was also affected by marker placement errors and the model scaling algorithm. Although we took carefully to place both virtual model markers and actual markers on participants at the same anatomical locations, kinematics had been shown to be highly sensitive to model marker locations (Lund et al., 2015). The locations of the markers at the knee joint axis more anterior resulted in a smaller knee flexion angle. There was an error in the placement of the markers on the pelvis on the body, resulting in the need to rotate the pelvic pitch angle to make the spatial position better match. The six models discussed in this article were developed to represent adults of varying heights so that the parameters of their bone geometries were not the same. The sizes of scaled models were not exactly the same, despite the scale algorithm’s best efforts to scale them.

In all studies involving muscle actuation simulations, including this one (Figure 4), obtaining simulated activations that exactly match the muscle’s measured sEMG remains an important challenge. Model M5 derived its muscle strength parameters from MRI data collected from healthy young people (Handsfield et al., 2014) rather than aged cadavers. However, the correlation coefficients of gastrocnemius muscle were one of the lowest among the different models. M6, among all models, produced the highest correlation coefficients between simulated muscle activation and measured rectus femoris sEMG. Its correlation coefficient standard deviations were the smallest, indicating that it had better performance in each subject. It modified muscle geometry and parameters, reducing the passive force of the hip and knee extensors. This improvement reduced the co-activation of antagonist muscles.

The results showed differences in kinematics, kinetics and muscle activation between the outputs yielded by the different models. Accurate modelling of muscles, bones and joints is important for consistency in joint kinematics, joint dynamics and muscle force estimation across different models (Mohout et al., 2023). In order to improve the accuracy of modelling, researchers measured precise joint anatomy using magnetic resonance imaging (MRI) (Valente et al., 2014) and calibrated muscle parameters by functional experiments (Garner and Pandy, 2003; Lund et al., 2015; Wu et al., 2016). In addition to model customization, there is potential for improvement in biomechanical analysis algorithms of locomotion. For the scale algorithm in Opensim, errors of the markers will cause joint kinematics to shift. These improvements may lead to more consistent results between generic musculoskeletal models.

In conclusion, this study provides valuable insights into the differences in biomechanical results obtained from different models. It highlights the importance of accurate modeling of muscles, bones, and joints for achieving consistency in joint kinematics, dynamics, and muscle force estimation. Further research can focus on exploring and addressing the variations between models and improving the algorithms used in biomechanical analysis of locomotion.

## 5 Conclusion

In our study, we conducted a comprehensive comparison of joint kinematics, kinetics, and simulated muscle activation results obtained from six commonly used models. The consistency of the kinematic results was similar across speed experiments, and the consistency of the kinematic results normalized by joint range decreased with increasing speed. The output values of the model showed the same characteristics in multiple subjects. Correlations between kinetic results from different models were stronger than those between kinematic results, and correlation coefficients for both kinetics and kinematics were greater than 0.85. The muscle activation yielded by RUN model and the activation yielded by Lai’s model were the most correlated with the sEMG signals of m. GAS and RF, respectively. We analysed the effects of model definition and parameter differences, the model scaling algorithm, and marker errors on the results. By quantifying the differences between the output results of different models, our research provides valuable insights for researchers in selecting the most appropriate model for their studies. Moreover, we believe that future research should focus on improving musculoskeletal models, developing methods for personalizing models, and advancing biomechanical analysis algorithms. These advancements will contribute to enhancing the accuracy and consistency of the results in this field.

## Data Availability

The raw data supporting the conclusion of this article will be made available by the authors, without undue reservation.
